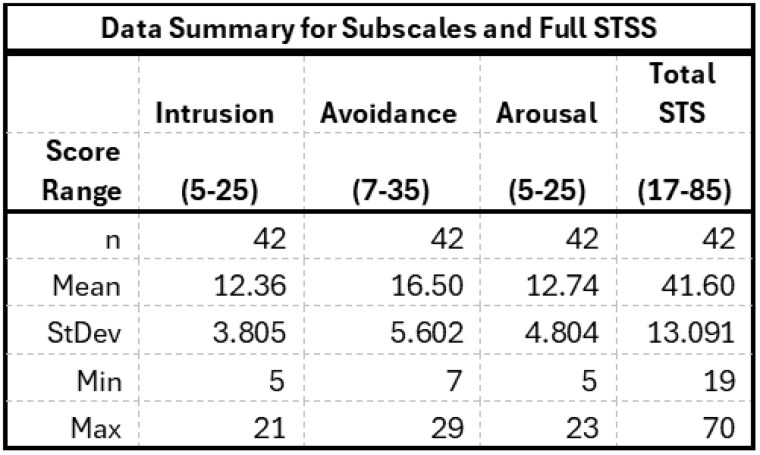# 637 Skin Deep? The Unseen Wounds of Burn Care Professionals

**DOI:** 10.1093/jbcr/iraf019.266

**Published:** 2025-04-01

**Authors:** Ashley Honea, W Michelle Spencer, Claudia Islas, Karen Richey, Kevin Foster

**Affiliations:** Diane & Bruce Halle Arizona Burn Center; Diane & Bruce Halle Arizona Burn Center; Diane & Bruce Halle Arizona Burn Center; Diane & Bruce Halle Arizona Burn Center; Diane & Bruce Halle Arizona Burn Center

## Abstract

**Introduction:**

Secondary Traumatic Stress (STS) is a consequence of caring for another individual who has themselves been exposed to a traumatic incident. The nature of burn injuries put burn healthcare staff at risk for developing STS as a result of caring for these trauma-exposed patients. STS symptoms mirror Post Traumatic Stress Disorder, manifesting in intrusion, avoidance, and arousal symptoms. STS research has initially focused on mental health professionals and has expanded to include other high-risk occupations. The purpose of this study was to determine the prevalence of STS among burn healthcare staff.

**Methods:**

The Secondary Traumatic Stress Scale (STSS) is a 17-item validated instrument used to evaluate the frequency of STS symptoms. The 5-point Likert scale is comprised of 3 subscales measuring intrusion, avoidance and arousal. The total score ranges from 17-85, scores are categorized as: no STS < 28, mild 28-37, moderate 38-43, high 44-48, severe ≥ 49. The scale was distributed to burn center professionals via RedCap.

**Results:**

Forty-two individuals responded to the survey, representing nearly 30% of invited personnel. The overall prevalence of STS was high with 38% scoring in the mild or moderate range and 43% scoring as high or severe.

**Conclusions:**

This survey was administered during our peak burn season, where we experienced an unusually large number of patients admitted whose injuries involved tragic circumstances. While this may have impacted our results, it highlights the alarming risk to burn professionals of developing STS. We recommend broader investigation using the STSS across burn centers to gain comprehensive insights and inform supportive strategies aimed at the development of resilience-related skills for team members. Fostering a resilient workforce is crucial for both maintaining high-quality care of our patients and a healthy work environment.

**Applicability of Research to Practice:**

This study highlights the unseen harm that burn professionals’ experience and emphasizes that therapeutic efficacy depends on burn healthcare professionals’ emotional self-regulation.

**Funding for the Study:**

N/A